# A Rare Case of Transposition of Great Arteries Uncovered With Progressive Rheumatic Aortic Stenosis

**DOI:** 10.1016/j.jaccas.2021.07.001

**Published:** 2021-08-19

**Authors:** Siddhant Yadav, Milind Phadke, Ajay Mahajan, Pratap Nathani

**Affiliations:** Department of Cardiology, Lokmanya Tilak Municipal General Hospital, Sion, Mumbai, India

**Keywords:** aortic valve stenosis, congenital heart disease, rheumatic heart disease, transposition of the great arteries, tricuspid valve, AS, aortic stenosis, IVC, inferior vena cava, LV, left ventricle, MV, mitral valve, PA, pulmonary artery, RA, right atrium, RHD, rheumatic heart disease, RV, right ventricle, TGA, transposition of great arteries, TV, tricuspid valve

## Abstract

The authors describe a case of transposition of great arteries with a large atrial septal defect with fusion of tricuspid valve leaflets and severe aortic stenosis. The latter two were likely rheumatic in etiology. The patient’s condition improved after atrial switch and aortic valve replacement surgery. (**Level of Difficulty: Intermediate.**)

## Central Illustration

**Figure:**
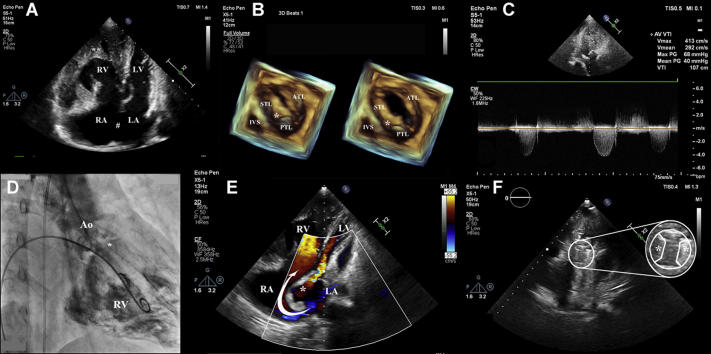

A 23-year-old man presented to us because of dyspnea on exertion and bluish discoloration of his lips. He was found to have cyanosis with pandigital and symmetrical clubbing of fingers and an oxygen saturation of 85%. A grade III ejection systolic murmur was heard in the right second intercostal space radiating to the carotids. The patient had received a diagnosis of a “heart defect” in childhood and had been advised to see a specialist. However, being only mildly symptomatic, he never followed up.

**Figure 1:**
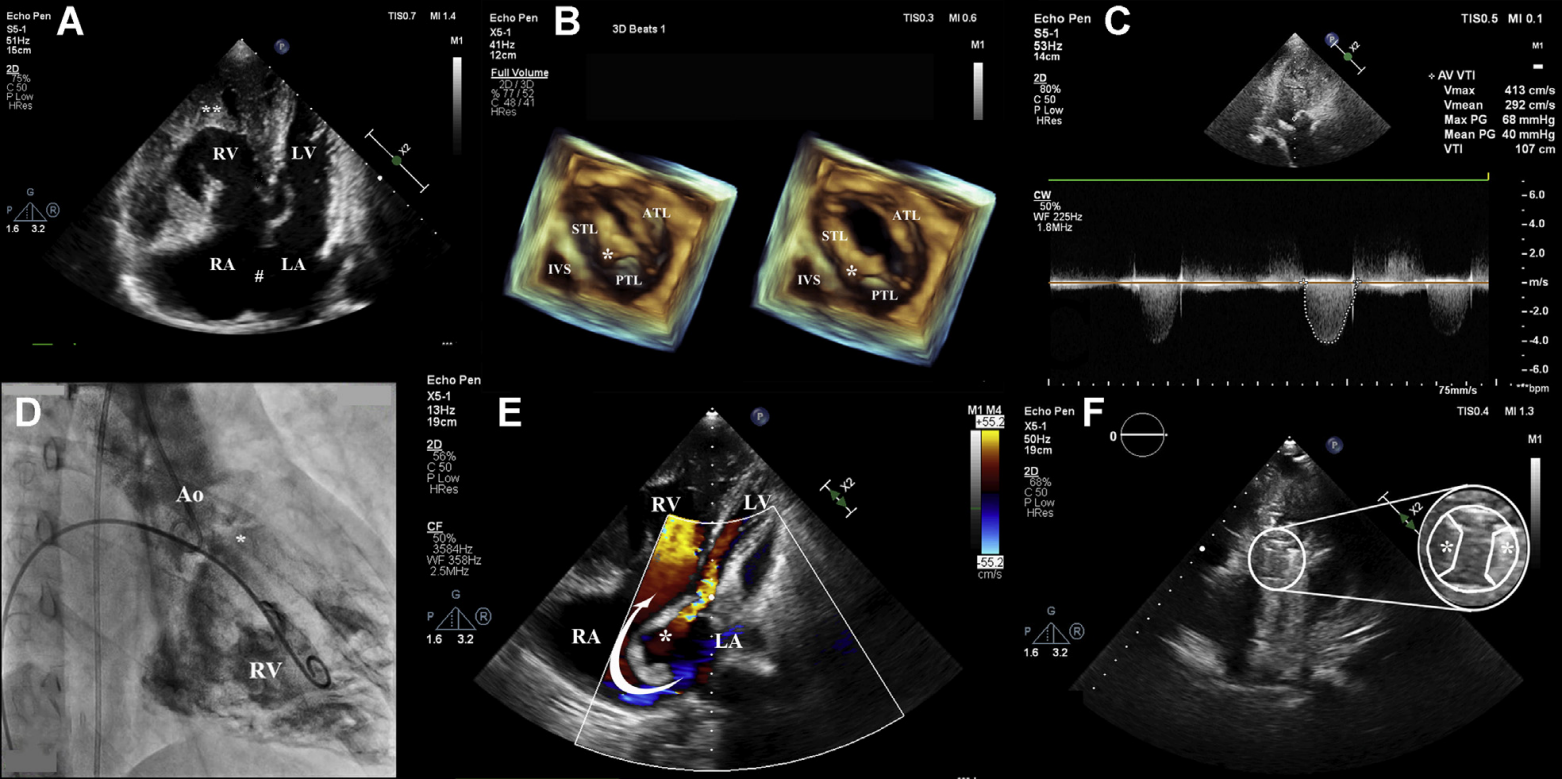
Echocardiographic and Angiographic Findings **(A)** Apical 4-chamber view showing a large ASD **(#)**. RV identified to the right owing to the increased trabeculations **(∗)** as compared to the LV. **(B)** 3D Zoom images of the tricuspid valve showing thickening and fusion of leaflets with raphe between them **(∗)**. **(C)** Doppler imaging of aortic valve showing severe AS. **(D)** Angiogram showing aorta arising from the RV and thickened aortic valve **(∗)**. **(E)** Postoperative color Doppler images showing the baffle **(∗)** and flow of blood from the LA to the RV **(arrow)**. **(F)** Demonstration of adequate disc excursion of the bileaflet **(∗)** prosthetic aortic valve. Also see Video 1. Ao = aorta; ASD = atrial septal defect; ATL = anterior tricuspid leaflet; IVC = inferior vena cava; IVS = interventricular septum; LA = left atrium; LPA = left pulmonary artery; LV = left ventricle; MPA = main pulmonary artery; PTL = posterior tricuspid leaflet; RA = right atrium; RPA = right pulmonary artery; RV = right ventricle; STL = septal tricuspid leaflet; SVC = superior vena cava.

Echocardiography showed two atria, with a large 36-mm ostium secundum atrial septal defect and bidirectional shunting of blood. The inferior vena cava (IVC) was seen draining into the right-sided morphologic right atrium (RA). The morphologic right ventricle (RV) and left ventricle (LV) were on their respective sides (Figure 1A, Video 1). There was mild systolic dysfunction of the RV. The interventricular septum was intact. The tricuspid valve (TV) appeared thickened, with almost complete fusion of its septal and posterior leaflets, typical of rheumatic heart disease (RHD) (Figure 1B, Video 1) (1). However, it showed no stenosis and only mild regurgitation on Doppler examination. The mitral valve (MV) was normal. The pulmonary artery (PA) and aorta arose from the LV and the RV, respectively; i.e., there was transposition of the great arteries (TGA). The aortic valve had 3 cusps, which were thickened and calcified, causing severe aortic stenosis (AS) (Figure 1C, Video 1). Cardiac catheterization confirmed the findings (Figure 1D, Video 1). Mean PA and pulmonary capillary wedge pressures were 30 mm and 15 mm Hg, respectively, making it likely that the mild pulmonary artery hypertension would improve after surgery.

Bicuspid aortic valve and RHD are the most likely causes of severe AS in young adults. The typical morphology of the TV, coupled with the high prevalence of RHD in India, made RHD the most likely cause. Primary TV disease in RHD is relatively rare, and the MV is generally affected concomitantly (1). Such affinity of RHD toward the mitral and aortic valves is thought to be due to the high pressures faced by them (2). However, in TGA the high pressures faced by a normal MV are faced by the TV. This possibly explains the tricuspid and aortic valve involvement in our patient without MV disease.

RV dysfunction is common in uncorrected TGA. The AS compounded this dysfunction. In cases of uncorrected TGA, the LV regresses quickly and cannot support the systemic circulation after 12 years of age. Although there are no clear recommendations for such a scenario, as per the 2018 adult congenital heart disease guidelines, owing to the patient’s age, atrial switch surgery was advised along with aortic valve replacement (3). On postoperative follow-up he was asymptomatic, with an oxygen saturation of 95%, and echocardiography showed adequately functioning baffle and aortic valve prosthesis (Figures 1D and 1E, Video 1).

Our case serves to highlight the rare entity of rheumatic AS in TGA and the peculiarity of tricuspid and aortic valve affection without MV disease in this case of RHD with TGA.

## Funding Support and Author Disclosures

The authors have reported that they have no relationships relevant to the contents of this paper to disclose.

## Appendix

Supplemental Video 1 Echocardiographic and Angiographic Findings
